# Online but not remote: Adapting field‐based ecology laboratories for online learning

**DOI:** 10.1002/ece3.7008

**Published:** 2020-11-21

**Authors:** Christoph F. Richter, Christopher J. Lortie, Tamara L. Kelly, Alessandro Filazzola, Krystal A. Nunes, Raani Sarkar

**Affiliations:** ^1^ Department of Biology University of Toronto Mississauga Mississauga ON Canada; ^2^ Department of Biology York University Toronto ON Canada; ^3^ Department of Biological Sciences University of Alberta Edmonton AB Canada

**Keywords:** data curation, field ecology, laboratories, online learning, open data collection, remote, undergraduate classes

## Abstract

Teaching ecology effectively and experientially has become more challenging for at least two reasons today. Most experiences of our students are urban, and we now face the near immediate and continuing need to deliver courses (either partially or wholly) online because of COVID‐19. Therefore, providing a learning experience that connects students to their environment within an ecological framework remains crucial and perhaps therapeutic to mental health. Here, we describe how prior to the pandemic we adapted our field‐based laboratories to include data collection, analysis, and interpretation, along with the development of a citizen‐science approach for online delivery. This design is simple to implement, does not require extensive work, and maintains the veracity of original learning outcomes. Collaboration online following field data collection in ecology courses within the context of cities offers further options to adapt to student experience levels, resource availability, and accessibility, as well as bringing instructors and students together to build an open well‐curated data set that can be used in ecology courses where no laboratories are available. Finally, it promotes an open collaboration among ecology instructors that can drive lasting conversations about ecology curriculum.

## Graphical abstract

Online laboratories in ecology cannot replace in‐person and field‐based instruction. However, when carefully designed, they can achieve the same learning outcomes and offer various options to adapt them for specific context. Importantly, they can provide opportunities for students to contribute to publicly accessible, long‐term databases, creating a network of ecology students, instructors, researchers, and the wider public.

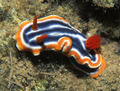

## 1 INTRODUCTION

Online learning is defined as the use of technology to either wholly or partially provide instruction and mechanisms for students to meet and collaborate (Moore et al., 2011). With recent advances in technology, adoption of online learning has expanded significantly in recent years (Canadian Digital Learning Research Association, 2019; Linder & Hayes, 2018; Major, 2016; Panigrahi et al., 2018) including teaching laboratory settings (e.g., Acosta et al., 2018) and teaching ecology is no exception.

With the COVID‐19 pandemic and associated restrictions, instructors across universities are in the process of adapting or redesigning their courses for partial or complete online and remote delivery. The changes required for this process depend on course content and online format (e.g., online synchronously/asynchronously, or as a hybrid with online and in‐person components) and are extensive for courses in the natural sciences that include laboratory‐based activities. For example, we teach large introductory ecology courses (150–400 students) with laboratories wherein students collect ecological data throughout the semester. Clearly, substantive changes must occur for the experiential nature of these courses to be maintained and course learning outcomes to be met. There is extensive literature regarding best practices in online teaching and to support this change in delivery format (Boettcher & Conrad, 2016; Linder & Hayes, 2018; Major, 2016; Nilson & Goodson, 2018), including science courses (Kennepohl, 2016).

Here, we instead focus on describing how redesigning our laboratories several years ago has helped move our courses, specifically laboratories, online while allowing us to meet our primary learning outcomes. We argue that a citizen‐science element and assignments based on scientific reporting allow our students to carry out most of the crucial elements of a practical, hands‐on laboratory in an online environment. We offer this as an example of how prior decisions were serendipitously beneficial and hope our example inspires further adaptations and redesigns. Indeed, the benefits of collaborative online element of field ecology, including data sharing, extend far beyond the immediate transition to online teaching.

## 2 ECOLOGY LABORATORY REDESIGN IN 2015–2016

### 2.1 Impetus to redesign

We teach large introductory ecology courses (~150–400 students depending on the year) on two suburban campuses. Most students are in their second year of university study with two general introductory biology courses supporting their learning thus far. Ecological instruction at this stage in the degree is crucial for two reasons. First, an increasing proportion of students are from an urban background, with little to no direct personal experience in semi‐natural or natural environments (Edwards & Larson, 2020; Li & Ernst, 2014). For instance, one student told one of us that he had never walked on unpaved paths before he began data collection in a field. Second, consistent anecdotal evidence from teaching second‐year ecology courses suggests that the students’ connection to natural systems is decreasing over time, which parallels a decline in interest and knowledge in natural history (Anderson, 2012; Tewksbury et al., 2014).

One of the goals of our courses is to provide students an opportunity to connect with an aspect of their surrounding that is likely new to most of them and typically does not form an important part of their daily experiences nor perceived emotional mindset. Indeed, learning in a natural setting is a logical choice for an ecology course because it provides numerous advantages. For instance, involvement in data collection and/or observations, practical activities that are integral parts of the scientific process, can increase students’ confidence and engagement with this process (Kloser et al., 2013) and help develop their data literacy by interacting with “authentic data” (Kjelvik & Schultheis, 2019). Similarly, working in field settings increases understanding of ecological concepts (Sheppard et al., 2010), as well as improve cognitive learning and performance (Easton & Gilburn, 2012). Learning in a field setting also helps increase self‐efficacy, GPA scores and graduation rates generally, but especially for students from under‐represented groups (Beltran et al., 2020). Furthermore, the impacts of learning in a field setting extend beyond academics and provide mental and physical benefits for our students (McKinnon et al., 2016; White et al., 2019).

Most students in our courses indicate in informal discussions that they do not intend to become ecologists. We assume that this may be partially due to the view that ecology is not relevant for urban living and that ecologists are mainly white males (O’Brien et al., 2020) working in remote and exotic field settings. We can correct this perception by highlighting the numerous connections and links between ecology and subjects that students find more personally relevant. In particular, the emerging field of urban ecology not only brings the activity of doing ecological field work from remote sites literally into students’ backyards, but it also highlights how ecological concepts, information, and models increasingly inform urban design and planning, architecture and communal policies (e.g., Grêt‐Regamey et al., 2017; see also About page of Journal of Urban Ecology, 2020). Thus, the value of the field components in our course is to demonstrate that ecology does not have to be done in a “far away field,” but instead connects our students to their local environment and to the scientific process.

### 2.2 Redesign

**Figure 1:**
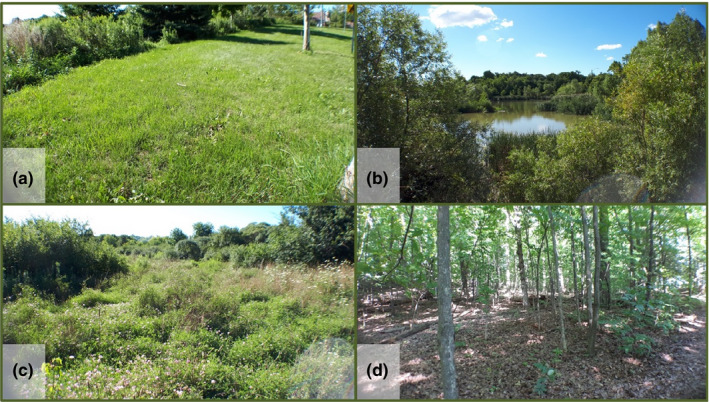
Four areas on campus that were used in the in‐person courses, exemplifying ecological diversity. a: Disturbed lawn, b: Wetland, c: Grassland, d: Forest

Using this framework, we redesigned our laboratory activities with four goals in mind (Table 1). We first wanted to give students a chance to experience their environment by leaving the confines of lecture halls and laboratory spaces. The type and intensity of learning changes based on the space in which it happens (Kirkby, 2014; Warkentin, 2011). By taking students out of their traditional learning spaces, and encouraging them to explore and collaborate with their peers in a range of on‐campus open‐air settings (Figure 1), we hoped the dynamic nature of their surroundings would inspire their curiosity and interest in the larger region in which they live, and to begin making connections with their own lives, backgrounds, and experiences (Barnes et al., 2019). While our laboratories do not specifically focus on natural history, the second goal was to gently introduce students to basic identification skills, providing a foundation for the development of basic natural history expertise and knowledge. Thirdly, we planned to give students a theoretical and practical introduction to the scientific research process including: formulating hypotheses, collecting data, and analyzing and interpreting the data in relation to their hypotheses. In particular, we wanted students to grasp the idea that science is a process and does not lead to final answers. By having students collect data and providing them with access to data from previous years, students become part of an authentic ongoing scientific process, ultimately illustrating how each individual contribution adds to a larger picture in the end (Kloser et al., 2013). Previous iterations of our laboratories demonstrated that students often do not pay much attention or care to their data collection if they know that the data will only be used for their course. By integrating their data into a larger public‐facing project, and, in turn, by giving students data from previous courses, the assignment takes on an authentic and experiential nature, which we hope will instill in students the importance of data reliability and quality, encouraging them to take responsibility and care for the information they collect and submit. Anecdotally, we and several of our colleagues have found that outward‐facing assignments—that is, those for which the products are shared with peers or the general public—generate higher quality work than those that are only for the consumption of the instructor. Finally, repeated measures of the same place through time will promote the importance of long‐term ecological data and engender a connection between current and past students as well as with students across the same city.

**Table 1:** Primary set of goals associated with a collaborative, online instruction ecology laboratory experience in Toronto in 2016 and 2017

Goal	Description	Learning outcomes	Online mechanism	Additional options
**1**	Get outside	· Appreciate and connect with natural spaces within the city. · Quantitatively explore biodiversity within the city	· iNaturalist, eBird, online identification tools and apps · Google Sheets to see and share data	Create accounts, get credit, and build observational data in globally shared tools
**2**	Introduce students to the field of natural history	· Develop basic identification and taxonomic skills. · Identify some local species of plants and animals.	iNaturalist, shared field notebooks, digital photographs, collaborative calls	Include real‐time Zoom or video‐conferencing calls with experts
**3**	Introduce students to the scientific process including experimental design principles for the natural sciences	· Formulate hypotheses and testable predictions. · Collect data in a structured manner consistent with rigorous experimental design principles. · Adapt and be flexible in design thinking in natural systems.	Version control, track changes, and online notebooks	Consider using a repository or formalized tracking tool for evidence including notes, data, and code such as GitHub
**4**	Publish data in publicly accessible repository	· Explain what data repository is and does. · Use a data repository. · Explain the function of metadata. · Write metadata accurately to contribute to a larger data set · Appreciate the value of collective, open collaboration.	· KNB Data Repository for compiled data · figshare for team data sets	Include students in deeper metadata writing and the process of compiling data across groups

Note

York University and the University of Toronto, Mississauga, collaborated to do the same field ecology laboratories. Students at each university did the same biodiversity data collection, independently.

Our fourth and last goal extends this idea of inspiring students to take data collection and maintenance seriously beyond their course‐related activities. Our plan is to compile data from across years and campuses into a large‐scale, long‐term database that is publicly accessible (Lortie et al., 2020). Thus far, our 3‐year collaboration between two universities in the same city has provided an opportunity to connect nearly 1,000 students with nature (and indirectly with each other), generating a data site comprising over 10,000 unique observations from transects or quadrats of the plants and animals in the city of Toronto. This is a considerable amount of data and adds to citizen‐science‐based approaches to study our environment. We have developed a data form for collection (see Appendix S1 for template) and are generating a set of detailed instructions (complete with collection protocol) that will be openly shared with faculty teaching introductory ecology classes and to the Ecological Research as Education Network (EREN; http://erenweb.org). The hope is that more classes from across Canada, North America and beyond will contribute to the database, thus offering an opportunity for larger‐scale analyses.

## 3 ADAPTING TO ONLINE LABORATORIES AND ECOLOGICAL EXPERIENCES

Clearly consuming educational content online is popular. The proportion of undergraduate students enrolled in distance or online courses increased from 16% in 2003/4 to 43% in 2015/16 (U.S. Department of Education, National Center for Education Statistics, https://nces.ed.gov/programs/digest/d18/tables/dt18_311.22.asp, accessed Aug. 21, 2020) and TED Talks were watched more than one billion times in 2012 (TED Blog, 2012). Shifting traditional lectures online is a relatively basic task of recording the live event and making that recording available online. However, maintaining active participation and building a learning community is much more difficult online than in‐person (Major, 2016; Nilson & Goodson, 2018) and is one of the major concerns for instructors either adapting or developing courses for online delivery under COVID‐19 restrictions (e.g., Arend, 2020; Stommel, 2020; Stone, 2020). The need to move field‐based ecological exercises online only adds to these complexities.

It is worth noting that we are focusing here on the adaptation of the design and instructional material of our laboratories to online teaching. This shift necessitates additional or different ways to support students while completing their laboratory work, such as online office hours, frequent monitoring of discussion boards, and additional TA support for these and other activities (e.g., Garrison & Cleveland‐Innes, 2005; Linder & Hayes, 2018; Major, 2016), as well as specific apps and simulations (e.g., Simbio, 2020). When used appropriately these tools will increase both instructor and student presence in the three domains identified by the community of inquiry model that guides online course development and promote virtual community (Boettcher & Conrad, 2016; Linder & Hayes, 2018). Here though, we focus on the specific case study of collaborative learning through experiential ecology laboratories (within and between universities).

With the required restrictions due to COVID‐19, in‐person laboratories will not be possible, at least not for all students. Even if a small number of students are able to attend laboratory sessions in person, most students will not be able to carry out laboratory work as we originally designed. The challenge therefore is to adapt these laboratories for a (mostly) online delivery while fulfilling most of our original learning outcomes (Table 2) and maintaining the benefits of field‐based learning discussed above. For this adaptation to the online environment, we divided the laboratories into two modules: data collection and analysis/interpretation.

**Table 2:** Steps to adapt laboratory to online delivery from a collaborative, remote format developed for a second‐year ecology course offering at two universities in the city of Toronto

In‐person laboratory	Adaptations for online laboratory	Alternative considerations	Advantage	Disadvantage
Introduction to hypothesis testing in tutorials	Introduction is delivered via recorded lecture or online session	Students read or view instructions and then meet online with TA to answer questions	–	–
In groups, students collect data in various locations across campus	Students collect data individually in locations accessible to them.	Written/video instructions include more detailed explanations and provide options for required equipment; protocol may be simplified by deleting some measurements altogether or reducing their frequency	Students collect own data, little change from in‐person laboratory	Students need to be able to access comparable areas; students may need to improvise if equipment is not available; data that are collected are not consistent with previous years
	Students collect data individually but contribute to group‐based worksheets	See above; may require additional time for group data entry and quality check	See above, plus group work is maintained	See above; effective communications within groups is essential and may require extra training/instructions
	Students use data from previous years	Instructions are edited to serve as background.	No additional requirements for students; predictable results	Students do not collect their own data; no opportunity for field experiences
Data analysis	Instruction for analysis is delivered via recorded lecture or online session	Additional resources may be needed, such as online office hours, links to online resources, discussion board postings, etc.	Because instructions are recorded, students have the opportunity to revisit	Troubleshooting is typically 1v1 reducing the potential of peer‐learning
Writing and submission of report	No change	–	–	–

### 3.1 Data collection

**Figure 2:**
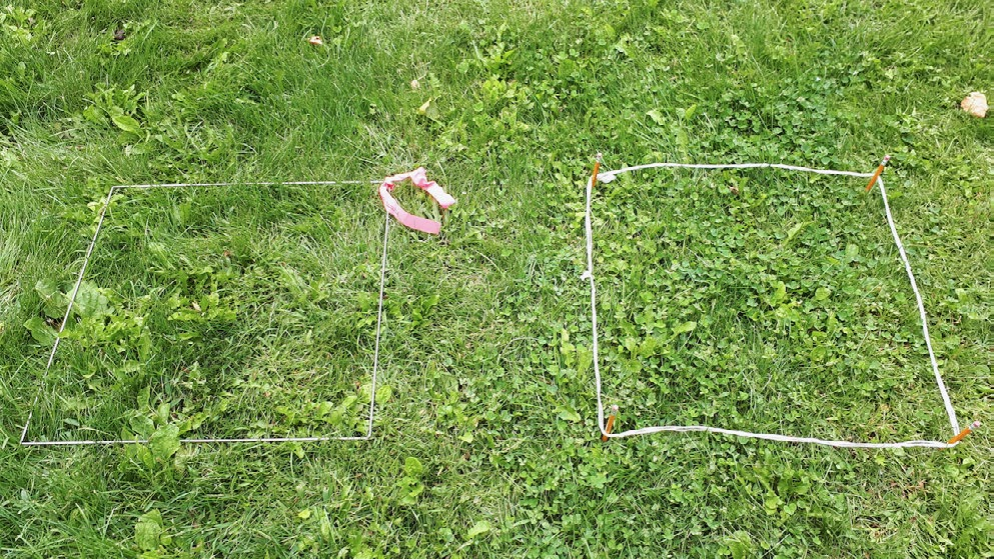
The left photograph shows a standard, commercially available, metal‐frame quadrat. On the right, a quadrat of equal size made with string and pencils as corner pegs

**Figure 3:**
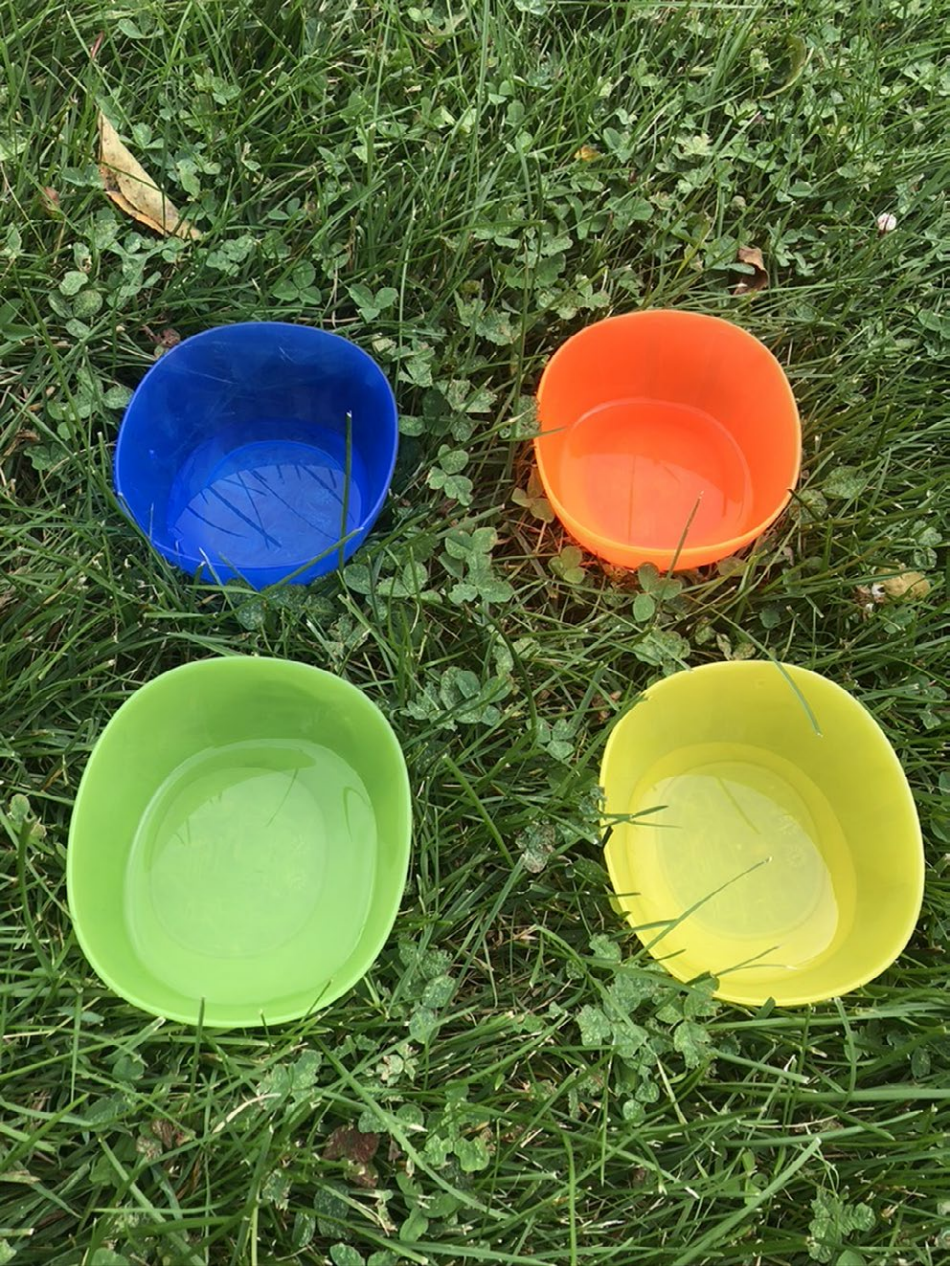
Small, colorful desert cups used as pan traps

The first module on data collection can be adapted in a variety of ways. First, for ecological and natural history data collection, students can sample their backyard, the patch of lawn in front of their apartment building, or a nearby park and use their phone GPS to provide location information. As not all of our students reside close to each other, the protocol must take into account the different climatic and geographical environments in which our students might be carrying out their measurements. Indeed, this “backyard” sampling would provide considerable heterogeneity to the data set, which can be a benefit (Kjelvik & Schultheis, 2019). Changes in instructions should address safety issues; as well, instructors should be aware of potential accessibility issues and be able to provide an alternative (e.g., observations via webcam such as https://explore.org/livecams). Because students will carry out this work on their own and thus will need more time for the activities, it may be necessary to reduce the amount or frequency of measurements. Methods must also be modified to not require specialized tools, or to suggest improvisations using common household items. For example, sampling done normally with quadrats, pan traps, etc., could be modified to:

- transects using measure tapes or butcher's string (with regular intervals marked using knots or marker)—for vertebrate, plant, and invertebrate sampling. Similarly, quadrats could be fashioned from strings and pencils (Figure 2).
- “stand and catalogue”—students would perform visual observations over a set period of time—vertebrate and plant sampling
- homemade pan traps, using any brightly colored container (e.g., margarine) filled with water and soap—for invertebrate sampling (Figure 3).

### 3.2 Data analysis/Interpretation

Because previous iterations have produced instructions on how to formulate a hypothesis and information on collection methods (via video and/or infographics) and data curation protocols (e.g., metadata), adapting the second module for online delivery does not require substantial changes. Instead, we focus our attention on providing more robust modes of explanation, such as videos that walk students through data curation and analyses.

Expansion of data analysis instruction in the online environment can provide a deeper learning experience for students. In fact, this part of the laboratory likely benefits from being online as students can revisit explanations as needed while working with their data. By highlighting additional resources and opportunities to reuse and learn from data in new ways in addition to direct ecological inquiry, we can spark student engagement, particularly for those with an interest in coding, computational biology, or data science (Lortie et al., 2020; Markowetz, 2017). This might open students up to a range of possible careers in environmental work.

### 3.3 Adaptability of protocol

For some online courses, data collection is not feasible, and this is where this protocol shows its adaptability. The adaptability of these laboratories is in the breadth of modifications—both as formative and summative assessments—that could be implemented. An alternative is to have students focus on analyzing data collected in previous years across both campuses or engaging in other activities. Students can still formulate hypotheses, test them against the data, and interpret their results. Along with data interpretation, students can be asked to improve the existing experimental design, to generate new instructions for different environments (or alternatively to produce more generalized instructions to promote adoption and application of the protocol by more schools). This would allow them to see creativity at work in the sciences, as well as the need for reproducibility (Reed, 2018). For some courses, a focus on data preparation for analysis might be warranted. For other courses with an emphasis on identification, students could identify plants by adding photos to the database, submitting some or all identifications for low‐stakes formative assessments. Through selective engagement with apps and other online tools that facilitate plant and animal identification, the barrier between technology and nature could be made more permeable; thus, encouraging students to use their phone in new ways to connect with nature.

This adaptability is evident for courses with and without data collection activities. Courses with an emphasis on ecological‐geographical interactions might include digital geographical resources, including Google Earth, The Global Biodiversity Information Facility, open data repositories, local GIS‐based information, local field guides, city maps and hiking resources, and other spatial resources to search out and select environments for novel data collection. Students could select two or three habitat types indicated on a local map featuring a GIS layer, and carry out their data collection in these habitats, promoting appreciation of georeferenced data for ecological research. Such an approach would enable groups of students to combine data from the same habitats, thus increasing their collective sample size. Alternatively, students could compare their data—both across space and time—with habitats in which they did not take measurements. The impact of the laboratory could be expanded by asking students to base their hypotheses on published data, or to compare their results with those from the existing literature. These are just a few suggestions to illustrate the adaptability of the online version of our laboratories.

We have worked to improve the laboratories each year for four years. To do this, we review feedback from teaching assistants and students (see Appendix S2 for an example of a survey) and test new ideas. With each iteration, we simplified and clarified data collection protocols to reduce cognitive load while ensuring students have enough time to complete all required steps while paying attention to detail. In an online environment, streamlined and focused instructions are critical to reduce students’ frustration and confusion while trying to collect data. The goal is to get students to connect with natural systems and collect data, not spend inordinate amounts of time in front of the screen reviewing complex instructions. This balance between enough detail and too little is critical for online ecology laboratories. We also worked to reduce the amount of questions students have while analyzing and interpreting the data thereby ensuring that students satisfy the proposed learning outcomes.

While adapting a field‐based laboratory to an online environment is not trivial we have provided some suggestions on how this could be done. A common thread through all these suggestions is that we need to pay special attention to design clear and unambiguous instructions and provide alternate ways of presenting them. For instance, adding recorded demonstrations can help clarify specific details or sequence of actions. On one hand, this will require more of our time and effort in the planning stage. On the other hand, such flexibility will facilitate access to our materials for students with different requirements by introducing Universal Design for Learning principles (Major, 2016; Rose et al., 2002).

The COVID‐19 pandemic was the specific impetus for us to develop our online ecological laboratories. Given the effort that went into the adaptation of the laboratories, and the various benefits and possibilities for further customization we outlined above, it begs the question whether these laboratories may not offer more than just a solution for a (hopefully) temporary problem. Are there reasons why we should continue to use these laboratories even once in‐person instructions is possible again? In this context, it is worth considering the evidence that virtual laboratories can be as effective as in‐person laboratories (Darrah et al., 2014), or even increase student achievement (Brinson, 2015). Also, online laboratories will likely use fewer resources and equipment, and thus be more cost effective. This argument is particularly salient for small institutions with limited budgets for laboratory improvements (Darrah et al., 2014). Importantly, this latter argument is only valid if students are not asked to supply equipment instead, effectively shifting costs from institution to students. Another benefit of online laboratories is that students can do their work at their own time, pace and location, making laboratories more accessible for students who have challenging work commitments or have difficulties attending in‐person classes (Grout, 2015).

Virtual laboratories facilitate access for some students; at the same time, they may be less accessible for others. Frequently, participation in these laboratories requires a stable internet connection. If this is not available, or attending online sessions or downloading required files increases costs, online laboratories may reduce students’ ability to engage with digital laboratory content. These issues could be partially solved by providing alternative formats with smaller bandwidth requirements (e.g., transcripts, compressed files, etc.) or that are fully accessible and functional on mobile devices. There is also the concern that taking online laboratories early on may prepare students less well for traditional, in‐person laboratories in higher courses (Faulconer & Gruss, 2018). One strategy to avoid this issue would be to align learning outcomes across years ensuring that online and in‐person laboratories focus on those learning outcomes that can be most effectively taught in each environment. Another potential limitation of virtual laboratories is that developing interpersonal relationships between students, and between students and instructors or teaching assistants, may become more difficult. One reason for this is the more scheduled and limited time to interact in an online setting (Faulconer & Gruss, 2018). Planning time to talk with each other, either at the end of scheduled periods, or as additional “office hours,” could help alleviate this concern. Also, inviting multiple students for group office hours similarly can support the development of relationships. A second reason for the lack of connection in online laboratories is our interactions are mediated through technology and lack the closeness of face‐to‐face meetings (Major, 2016). Careful choice of tools and platforms on which to run laboratories and that facilitate interactions could reduce these feelings of removedness. Additionally, selecting activities that allow students to connect and collaborate would further help them to get to know each other and to form effective relationships (Major, 2016).

## 4 FUTURE WORK

We are currently assessing the impact of the above‐described laboratories on student understanding of the scientific process and their approach on data collection and maintenance. We are still exploring the most effective and sustainable way to make the data available to the wider scientific (including other universities’ ecology courses) and non‐scientific public, while adhering to university regulations (Brinson, 2017). Ideally, this would be as a dedicated, open database, to which any course who follows our established protocols could contribute. Alternatively, we could contribute to already existing platforms and use version control from year‐to‐year to update the same data asset in the repository through time.

Teaching ecology pragmatically and hands‐on remains, in our view, an important component of undergraduate biology programs, not only for the knowledge it provides but for the benefit of providing students with an opportunity allows students to connect with the environment they live and work in. This is difficult enough to accomplish in “normal,” in‐person settings, but is now even more challenging given delivery online and uncertainty about how long restrictions are in place. The introductory ecology laboratories we adapted here can provide experiences and learning opportunities that are comparable to in‐person instruction and address our original learning outcomes. They also provide a sustainable framework for instructors due to their adaptable structure and straight‐forward implementation. Finally, they extend the impact of our teaching and student work beyond campus and academia, offering an opportunity for collaboration between students, researchers from different universities, and their communities.

## CONFLICT OF INTEREST

None declared.

## AUTHOR CONTRIBUTIONS

**Christoph Richter:** Conceptualization (lead); writing–original draft (lead); writing–review and editing (equal). **Christopher J. Lortie:** Conceptualization (lead); writing–original draft (equal); writing–review and editing (equal). **Tamara Kelly:** Conceptualization (lead); writing–original draft (equal); writing–review and editing (equal). **Alex Filazzola:** Conceptualization (equal); writing–original draft (equal); writing–review and editing (equal). **Krystal A. Nunes:** Conceptualization (equal); writing–review and editing (equal). **Raani Sarkar:** Conceptualization (equal); writing–review and editing (equal).

## Supporting information

Appendix S1‐S2

## Data Availability

Data collected in the courses are available at Christopher J Lortie, Alessandro Filazzola, Christoph Richter, and Tamara Kelly. 2020. Campus ecology network biodiversity data: York University and The University of Toronto Mississauga. knb.ecoinformatics.org/view/doi:10.5063/F1CN728G.
