# Supplementary material for: Online but not remote: Adapting field‐based ecology laboratories for online learning
Source: Ecol Evol. 2020 Nov 21;11(8):3616–24. doi: 10.1002/ece3.7008 (PMC8057323; doi:10.1002/ece3.7008)
Supplement: Supplementary file 1 — Appendix S1‐S2 [file ECE3-11-3616-s001.docx]

### Appendices

Appendix 1: Example of a data form used by students to collect data in the field

Campus Community Structure & Biological Diversity – Data Collection Sheet

*(Completed Excel Spreadsheet Submission - Total Marks: 2%*)

- This data collection sheet will be completed as a group.
- Make sure that you STAY ON THE TRAILS to AVOID TRAMPLING/DAMAGING the vegetation.
- A final excel DATA SUBMISSION is due before the start of the next lab.  The excel spreadsheet will include additional columns that need to be correctly filled out in order to get full marks.

| Habitat: |  | Flag #: |  |
| --- | --- | --- | --- |
| Plant Identification | # | Invertebrate Identification | # |
| 1. |  | 1. |  |
| 2. |  | 2. |  |
| 3. |  | 3. |  |
| 4. |  | 4. |  |
| 5. |  | 5. |  |
| 6. |  | 6. |  |
| 7. |  | 7. |  |
| 8. |  | 8. |  |
| 9. |  | 9. |  |
| 10. |  | 10. |  |
| TOTAL # of individual plants: |  | TOTAL # of individual invertebrates: |  |

| Habitat: |  | Flag #: |  |
| --- | --- | --- | --- |
| Plant Identification | # | Invertebrate Identification | # |
| 1. |  | 1. |  |
| 2. |  | 2. |  |
| 3. |  | 3. |  |
| 4. |  | 4. |  |
| 5. |  | 5. |  |
| 6. |  | 6. |  |
| 7. |  | 7. |  |
| 8. |  | 8. |  |
| 9. |  | 9. |  |
| 10. |  | 10. |  |
| TOTAL # of individual plants: |  | TOTAL # of individual invertebrates: |  |

Appendix 2: The following questions are part of a survey to obtain feedback from students about the lab activities at the end of the semester. Questions in bold use a five-point Likert-scale (Strongly agree – strongly disagree).

This survey allows you to evaluate and provide feedback for the practical component of the course.  Please note that your answers will be anonymous and neither the TAs or course instructor will review any of the comments until after your final grades have been submitted to the registrar (so any feedback you give us can in no way negatively affect your grade).  Your honest input is greatly appreciated, and it helps us to improve the practical experience for future students.

Thank you.

1. **I enjoyed the content and practical skills used in Practical 1 – Campus Diversity Preliminary Observations.**
2. Please provide additional comments or suggestions about Practical 1 (e.g., What did you like most/least?  Is there anything you would change to improve the experience?)
3. **I enjoyed the content and practical skills used in Practical 2 – Campus Diversity Data Collection.**
4. Please provide additional comments or suggestions about Practical 2 (e.g., What did you like most/least?  Is there anything you would change to improve the experience?).
5. **I enjoyed the content and practical skills used in Practical 3 – Literature Critique.**

1. **Practical 3-Literature Critique helped me learn how to critically read and understand scientific literature.**
2. **Practical 3-Literature Critique was valuable in learning how to write my own formal introduction and formal results sections.**
3. Please provide additional comments or suggestions about Practical 3-Literature Critique (e.g., What did you like most/least?  Is there anything you would change to improve the experience?).
4. **I enjoyed the content and practical skills used in Practical 4 – Soil Collection & Diversity Data Analysis.**
5. **The data analysis tutorial during Practical 4 was very helpful for completing the formal results and discussion assignment.**
6. Please provide additional comments or suggestions about Practical 4-Soil Collection & Campus Diversity Data Analysis. (e.g., What did you like most/least?  Is there anything you would change to improve the experience?)
7. **I enjoyed the content and practical skills used in Practical 5 – Soil Analysis.**
8. Please provide additional comments or suggestions about Practical 5-Soil Analysis (e.g., What did you like most?  Is there anything you would change to improve the experience?
9. **I enjoyed the integrated structure of the Campus Diversity & Soil Analysis activities and associated assignments (i.e., the fact that all of the activities were related to each other).**
10. In the fall term, students were required to write a formal lab report based on the class campus diversity data (collected in a similar way to the field activity you participated in during Practical 2).  For this activity, each student developed their own unique hypothesis and determined which data and statistical tests they should use to complete their formal report.  Considering how the campus diversity activities (and associated assignments) were run in the fall and summer terms, which of the following statements best describes how you think these activities should be run in the future?
    1. I think each student should be able to come up with their own unique hypothesis and figure out which data and statistical tests need to be used for their formal report/assignments (with limited guidance from their TAs).
    2. I think each student should be able to come up with their own unique hypothesis and figure out which data and statistical tests need to be used for their formal report/assignments (with significant guidance from their TAs).
    3. I think students should be given a list of hypotheses to choose from for their formal reports/assignments, with specific data analysis instructions for each.
    4. I think all students should be given the same general hypothesis and specific data analysis instructions for their formal report/assignments.
    5. I would keep the activity and assignment structure of the Summer 2019 course (e.g., some flexibility in writing the formal introduction but specific instructions for data analysis and results).
11. Please provide additional comments/suggestions for your answer to the previous question.
12. **Overall, the BIO205 practicals were worthwhile and added to my enjoyment of the course and my interest in ecology.**
13. Please provide any additional comments or suggestions about the BIO205 practicals (i.e., anything you think was not adequately addressed in the previous questions).
